# Supplementary material for: Imatinib Optimized Therapy Improves Major Molecular Response Rates in Patients with Chronic Myeloid Leukemia
Source: Pharmaceutics. 2022 Aug 12;14(8):1676. doi: 10.3390/pharmaceutics14081676 (PMC9414005; doi:10.3390/pharmaceutics14081676)

Supplemental materials

Supplemental Table S1. Safety profile of the OPTIM-imatinib study.

**All adverse events N and % of all** Recurrent events were counted once	**TDM arm**						**Control arm**						**Observational arm**					
	All		G1-2		G3-4		All		G1-2		G3-4		All		G1-2		G 3-4	
	N	%	N	%	N	%	N	%	N	%	N	%	N	%	N	%	N	%
Asthenia	**8**	*23*	6	*19*	2	*5*	**2**	*9*	2	*9*	0	*0*	**4**	*9*	4	*9*	0	*0*
Blood cell counts	**6**	*14*	3	*7*	3	*7*	**9**	*21*	6	*14*	3	*7*	**8**	*17*	5	*11*	3	*6*
Cancer	**0**	*0*	0	*0*	0	*0*	**1**	*2*	0	*0*	1	*2*	**1**	*2*	0	*0*	1	*2*
Cardiovascular	**2**	*5*	2	*5*	0	*0*	**3**	*7*	3	*7*	0	*0*	**7**	*15*	5	*11*	2	*4*
Edema and fluid retention	**5**	*12*	3	*7*	2	*5*	**5**	*12*	5	*12*	0	*0*	**13**	*28*	12	*26*	1	*2*
Eyes symptoms	**2**	*5*	2	*5*	0	*0*	**2**	*5*	1	*2*	1	*2*	**0**	*0*	0	*0*	0	*0*
Gastro-intestinal symptoms	**14**	*33*	13	*30*	1	*2*	**5**	*12*	5	*12*	0	*0*	**14**	*30*	13	*28*	1	*2*
Hemorrhage	**0**	*0*	0	*0*	0	*0*	**0**	*0*	0	*0*	0	*0*	**2**	*4*	1	*2*	1	*2*
Infections	**1**	*2*	1	*2*	0	*0*	**3**	*7*	2	*5*	1	*2*	**6**	*13*	3	*6*	3	*6*
Metabolic abnormalities	**5**	*12*	2	*5*	3	*7*	**2**	*5*	2	*5*	0	*0*	**5**	*11*	1	*2*	4	*9*
Musculoskeletal symptoms	**9**	*21*	9	*21*	0	*0*	**3**	*7*	3	*7*	0	*0*	**10**	*21*	9	*19*	1	*2*
Neurological	**5**	*12*	5	*12*	0	*0*	**2**	*5*	2	*5*	0	*0*	**2**	*4*	2	*4*	0	*0*
Pain, other	**1**	*2*	1	*2*	0	*0*	**0**	*0*	0	*0*	0	*0*	**1**	*2*	1	*2*	0	*0*
Skin, mucosa	**9**	*21*	9	*21*	0	*0*	**9**	*21*	7	*16*	2	*5*	**9**	*19*	9	*19*	0	*0*
Transaminases and GT	**2**	*5*	0	*0*	2	*5*	**4**	*9*	3	*7*	1	*2*	**1**	*2*	0	*0*	1	*2*

Supplemental Figure S1

All adverse events categories representing more than 10% of the patients are represented. In this radar graph, the black line represents the safety profile of the control arm as compared with the TDM arm (dashed line) and the observational arm (grey line).

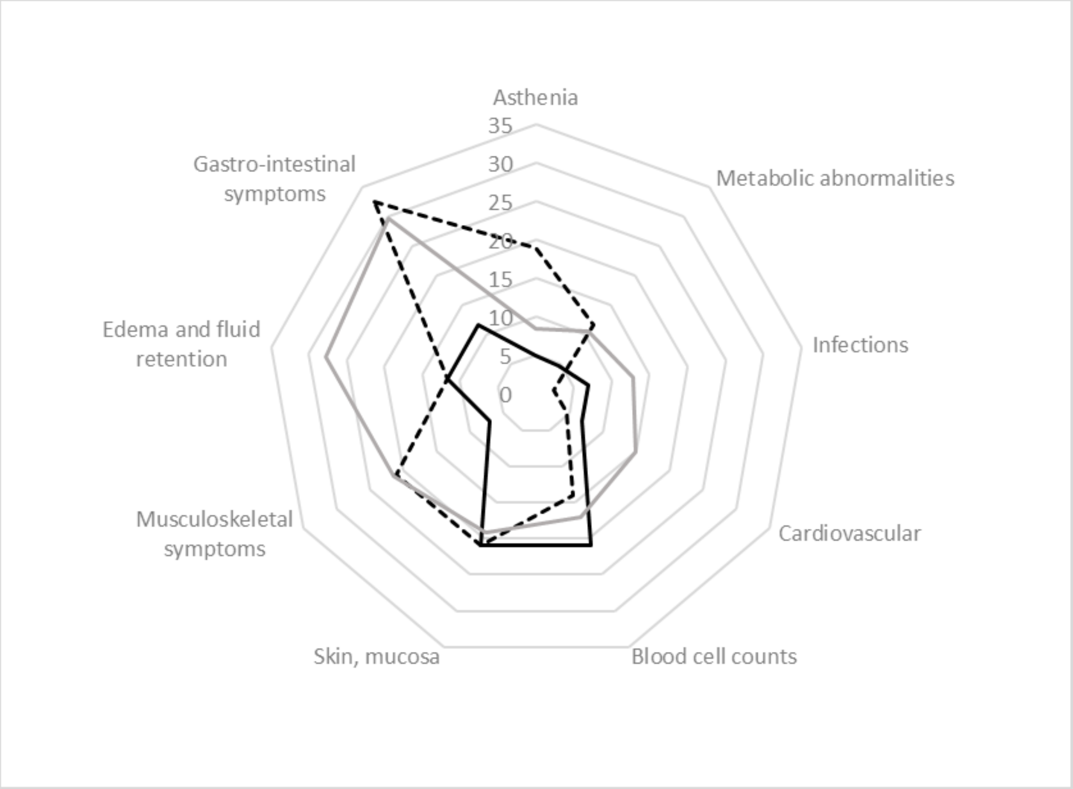

Supplement: Supplementary file 1 [file pharmaceutics-14-01676-s001.zip › pharmaceutics-1817957-supplementary.pdf]
